# A System of Midwifery

**Published:** 1876-03

**Authors:** 


*A System of Midwifery, Including the Diseases of Pregnancy and the Puerperal State.* By WILLIAM LEISHMAN, M. D. Second American from the Second Revised English Edition. With Additions by JOHN S. PARRY, M. D. Philadelphia : Henry C. Lea. 1875. Buffalo: T. Butler & Son.

The first edition of this work having become exhausted both in England and the United States, the author has prepared a second, which he has carefully revised and corrected. The writer has given greater prominence to the physiological questions involved in the study of midwifery than in the first edition. The chapters on puerperal fever have been re-written to give prominence to the theory of septicæmia, which the has adopted since the last edition.

The chapters upon the forceps in the former edition were not in accord with the more generally accepted views of American physicians, and hence the Ameri con editor, Dr. Parry, has added much to this section of the work. This portion as it now stands, is a very acceptable exposition of the subject. Lactation and and the puerperal diseases have also received attention from Dr. Parry, who has added to the work a chapter on Diphtheria of Puerperal Wounds, together with some new illustrations. The work of the American editor is exceedingly well done, and is an addition of great value to the work.

As we write the above, news comes to us of the death of Dr. Parry, in Jacksonville, Florida, in his thirty-fourth year. Dr. Parry had spent two previous winters in Florida for the benefit of his health. In order to prepare the work under consideration, and his own monograph upon Extra-uterine Pregnancy, for the press, he remained in Philadelphia until December. We do not know that such is the fact, but surmise that the task was too great for one in his condition of health, and he only hastened his death by his devotion to work.

